# A Systematic Review of Worldwide Guidelines, Protocols, and Health Care Standards for Provision of Palliative Care Services in Progressive Neurological Disorders

**DOI:** 10.1111/jnu.70130

**Published:** 2026-09-08

**Authors:** Jessica Kaplonyi, Samantha M. Loi, Julie C. Stout, Jennifer Philip, Rosalie Gebert, Anita M. Y. Goh

**Affiliations:** ^1^ Faculty of MDHS The University of Melbourne Parkville Victoria Australia; ^2^ Neuropsychiatry Centre The Royal Melbourne Hospital Melbourne Victoria Australia; ^3^ School of Psychological Sciences and the Turner Institute for Brain & Mental Health Monash University Melbourne Victoria Australia; ^4^ Department of Medicine St Vincent's Hospital Melbourne Victoria Australia; ^5^ Independent Researcher Parkville Victoria Australia; ^6^ National Ageing Research Institute Melbourne Victoria Australia

**Keywords:** clinical practice guidelines, end‐of‐life, guideline, palliative care, progressive neurological disease, systematic reviews

## Abstract

**Background:**

Progressive neurological disorders (PNDs) are life‐limiting conditions with complex trajectories. PNDs require holistic, person‐centred care that responds to the needs of both individuals and their families, including the provision of palliative and end‐of‐life (EOL) care.

**Aims:**

This systematic review aimed to identify and synthesize existing international guidelines addressing palliative and EOL care in adult PND populations, with particular attention to their quality and scope.

**Methods:**

We searched databases and gray literature sources for guidelines published between January 2013 and February 2026. Thirty‐three guidelines were included for review. We appraised guidelines to assess quality and comprehensiveness against the World Health Organization's (WHO) domains of palliative care. We conducted inductive content analysis to identify key themes.

**Results:**

Guidelines predominantly were published from high‐income countries and focused on dementia. Overall guideline quality was high. Most addressed physical, psychosocial, and social dimensions of care of the WHO domains; however, spiritual wellbeing was inconsistently addressed. Six interrelated themes emerged: (i) holistic symptom management; (ii) recognition of families and caregivers as partners in care; (iii) early and ongoing communication; (iv) value‐aligned decision‐making; (v) multidisciplinary team expertise; (vi) navigation of medicolegal complexities.

**Conclusion:**

Findings indicate broad international consensus on the principles of high‐quality palliative care in PND, while highlighting gaps in disease/diagnosis representation, spiritual care, and global equity. Future research could consider the importance of disease‐specific guidelines, covering regions and countries from across a range of high‐, middle‐, and lower‐income countries.

**Clinical Relevance:**

Clinicians caring for people with progressive neurological disorders can draw on the internationally consistent principles identified in this review including early integration of palliative care, proactive and open communication, and recognition of families as partners in care to benchmark and strengthen their practice. Gaps in spiritual wellbeing, condition‐specific guidance, clinical supports, and equitable access represent clear targets for workforce education, service development, and health policy advocacy.

## 1 Introduction

Progressive neurological disorders (PNDs) are chronic degenerative conditions of the nervous system that progressively impair activities of daily living for affected individuals (BISA 2025; Elamri et al. 2025). PNDs lead to a combination of movement, cognitive, and psychological impairments, caused by the gradual loss of neurons. Diagnoses include conditions that result, or may result, in dementia Parkinson's disease, Amyotrophic Lateral Sclerosis, Huntington's disease, and the subtypes of dementia including Alzheimer's disease. Most PNDs have no cure and vary widely in etiology, symptom profile, and disease trajectory. Care requires a holistic, multidisciplinary approach encompassing symptom management, psychosocial, psychological, and spiritual care. As PNDs are life‐limiting and associated with premature death, palliative care is a core component of optimal care.

Palliative care is a team‐based approach that aims to improve quality of life and prevent suffering through early identification of needs and person‐centered support (Boersema‐Wijma et al. 2023; Palliative Care Australia 2023; World Health Organization (WHO) 2020). It is distinct from end‐of‐life (EOL) care, which is provided when a person's death is imminent (Teoli et al. 2025). People with PND and their families face unique disease‐related burdens, arising from variable survival rates, unpredictable symptom progression, and evolving family relationships as caregiving responsibilities increased. (Decruyenaere et al. 2005; Kinchin et al. 2025; Mahmood et al. 2022). This represents a significant public health challenge, as the burden extends to families and health systems, with many healthcare providers lacking confidence, training, and support to manage the practical and emotional complexities of PND care, including of palliative and EOL care (Aubeeluck 2005; Bos et al. 2019; Lerum et al. 2017; Lorenz et al. 2006; Wilson et al. 2011).

Care needs are often complex from the time of diagnosis. As such, there is a clear need to for timely, high‐quality palliative care to support physical comfort, psychological wellbeing, spiritual needs, family involvement, and caregiver health and wellbeing. Early introduction of palliative care can assist with smooth transitions between disease stages and future planning, which may in turn reduce the burden on families and patients (Durepos et al. 2017; Wang et al. 2022; WHO, n.d.). Carers and family members often play a crucial role in decision‐making and providing support throughout the disease process, with a known relationship existing between patient symptoms and reports of caregiver burden (Chiao et al. 2015; Perez et al. 2022; Pickett et al. 2007).

Currently, there is substantial variation in how palliative care is defined, delivered, and standardized across health systems worldwide. Guidelines, protocols, and healthcare standards exist for palliative care in PND and provide healthcare professionals with best practice principles to guide their practice.

To our knowledge there has been no analysis of their scope, quality, and comprehensiveness. This systematic review aims to identify and examine existing worldwide guidelines related to the provision of palliative care in PND. Specifically, it seeks to determine the nature of current recommendations; explore which domains of health and wellbeing they cover; and identify common themes or divergences across documents. By comprehensively appraising and synthesizing this body of guidance, this review contributes to a clearer understanding of the global landscape of palliative care standards in PND and supports future improvements in policy, practice, and patient‐centred care.

We expected recommendations to be diverse based on their origin from diverse sources, such as government bodies and health organizations, to professional associations and gray literature, each contributing differently to the evidence and practice environment. We hoped that by synthesizing existing work in this area, we could identify opportunities for harmonizing, standardizing, and eventually improving recommendations.

## 2 Design

This systematic review followed the Preferred Reporting Items for Systematic Reviews and Meta‐Analysis (PRISMA) reporting guidelines. We developed a protocol and pre‐registered on Prospero: CRD42023393759: PROSPERO. The full list of search terms is available in Table S1.

## 3 Materials and Methods

### 3.1 Identification and Selection of Papers and Their Recommendations

Included policies or guidelines were those published by a government body or a private or public healthcare service, contained guidance or suggestions for provision of palliative care for people with PND, within the adult population, were current documents/versions, and were published within the last 10 years (January 2013–January 2023 inclusive). We conducted the initial search on 5/8/2023 with an additional search conducted on 2/2/2026 for new publications since January 2023.

The inclusion criteria above were designed to optimize credibility and reliability, relevance to policy and practice and to minimize bias. Government bodies and healthcare services (public or private) were considered authoritative sources, as their outputs are more likely to adhere to rigorous methodologies, ethical standards, and peer‐review processes, thereby enhancing the trustworthiness of the data. Studies from these sources were also chosen to reflect real‐world healthcare practices, policies, and outcomes, and their inclusion was to optimize that the findings are applicable to healthcare decision‐making and policy development. By focusing on studies from recognized institutions, we aimed to reduce the risk of bias that might be present in less formal or non‐peer‐reviewed sources.

Exclusion criteria included papers not in English, pediatric populations, and non‐neurodegenerative palliative conditions (e.g., cancer, heart disease, organ failure, mental illness).

### 3.2 Search Strategy

The PICOC formulation (population, intervention, comparator, outcome, context) guided the refinement of the research question. The research questions and comprehensive search strategy were developed with the assistance of a specialist medical librarian who provided guidance on keyword selection and search methodology. The initial database searches were conducted on 5/8/2023, within: Medline, PubMed, Web of Science, CINAHL, Scopus, PsycINFO, TRIP database, Cochrane Library, ProQuest Central, RNAO. The search was repeated on 2/2/2026 to include new guidelines published since the initial search.

**FIGURE 1:**
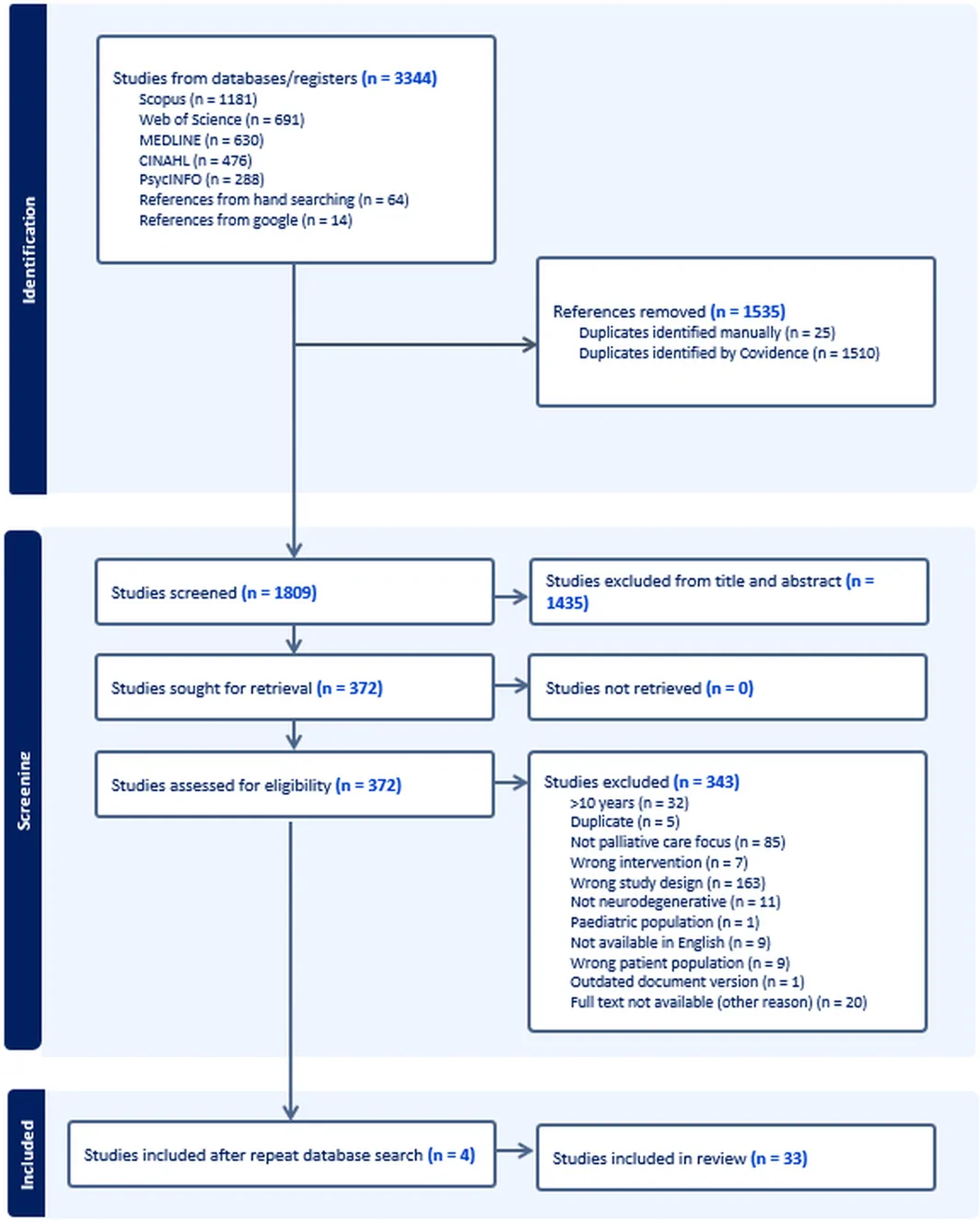
PRISMA flow diagram illustrating the identification, screening, eligibility, and inclusion of records.

Detailed search strategies are listed in Table S1. The searching, screening, and selection process is summarized in Figure 1.

Covidence was used to assist title and abstract screening, which was independently screened by two authors. Full texts were then co‐reviewed by two authors, who collaboratively reviewed for eligibility according to the predetermined criteria, working together to reach consensus on study selection. Conflicts were independently evaluated and arbitrated by a third author.

We manually searched the reference lists of included full‐text articles to identify any additional relevant studies that may not have been captured in the initial database searches, and we conducted a Google search of simplified terminology and keywords of the first 10 pages of results, as agreed upon by the research team.

### 3.3 Data Management and Extraction

The research team collaboratively developed and tested a standardized data extraction tool to ensure consistent and accurate data collection. For the included studies, one reviewer read each guideline in full and extracted key information about each paper, including the country of origin, disease focus, total number of recommendations, and number of palliative/EOL recommendations. In determining exactly what was a recommendation, attention was paid to instructive terminology such as “should”, “must”, “best practice”, “essential”, and “important”.

### 3.4 Quality Assessment

We assessed the integrity and methodological rigor of included studies to ensure the reliability and objectivity of the quality assessment process, enhancing the overall validity of the review. The quality of each publication and risk of bias was appraised using the AACODS (Authority, Accuracy, Coverage, Objectivity, Date, Significance) Checklist (Tyndall 2010). The AACODS framework provides a systematic way to assess these sources across six key domains, where some domains are further defined by additional quality parameters: *Authority* (who produced it), *Accuracy* (how reliable the information is), *Coverage* (scope and completeness), *Objectivity* (presence of bias), *Date* (timeliness), and *Significance* (relevance and impact).

Designed specifically to evaluate and critically appraise gray literature, the AACODS Checklist (see Supporting Information). has been used extensively internationally (Bandyopadhyay et al. 2020; Clark et al. 2022; Jatau et al. 2021; Khosravi et al. 2025; Xiong et al. 2024) and we selected it given the diversity and variability of gray literature identified as guidelines and policies. J.K. conducted the initial appraisal, with a sample of 10 (32% of the included full text papers) randomly selected publications also reviewed against the same checklist by a second assessor who ensured consistency of ratings. If there was a difference, both reviewers collaboratively discussed discrepancies until the reviews were consistent. Major discrepancies in the scores (where assigned scores differed by more than two points) were also discussed and independently reassessed by another author until consensus was achieved. For the *Date* domain—which asks the date, whether the date can be ascertained, and whether key contemporary material was referenced—if the first two parameters were answered “no”, an overall negative result was applied for this domain.

### 3.5 Assessment of Holistic Care

We assessed each paper against the four domains of palliative care as defined by the World Health Organization (World Health Organization 2004). This framework defines palliative care as addressing physical, psychosocial, and spiritual problems and supports consistent, holistic care across services, settings, and roles, guides service development, workforce capability, and quality improvement. Use of this framework enabled a consistent and systematic appraisal of the extent to which the reviewed literature reflected a comprehensive, holistic approach to care. Importantly, the WHO domains are internationally recognized and applicable across care settings, professional roles, and service models, making them well suited to evaluating care in the context of PND. The framework also provides a shared language for service development, workforce capability building, and quality improvement, and aligns practice with national and international palliative care standards. As such, applying the WHO framework strengthened the rigor of the review and ensured that analysis was grounded in an established, person‐centred model of palliative care. We mapped guideline recommendations to the four WHO domains of palliative care, and we assessed the extent and depth of coverage within each domain to identify areas of alignment, inconsistency, and omission. The four domains are:

1. Physical Wellbeing: Pain assessment, dyspnoea assessment, delirium assessment, nutrition assessment, and oral health assessment.
2. Social and Occupational Wellbeing: practical concerns around family supports and considering a person's life story when providing care.
3. Psychosocial Wellbeing: Regular assessment of a person's mental health, particularly anxiety and depression around death and dying.
4. Spiritual Wellbeing: Regular assessment of a person's religious, spiritual, cultural, and traditional needs.

### 3.6 Synthesis of Guideline Recommendations and Content Analysis

We employed inductive content analysis to analyze the scope, content, context, and consistency of the guidelines, including areas of alignment and divergence in messaging. The first author used NVivo and principles of inductive content analysis to assign codes to the recommendations and ideas within each guideline (Braun and Clarke 2022). The labels were flexible and dynamic, with additional labels developed iteratively throughout the analytical process as data items were compared (Kleinheksel et al. 2020; Vears and Gillam 2022). Through the process of reflexivity and constant comparison, codes were then grouped into themes which aimed to be representative of the whole data set. However, the homogenous nature of many of the recommendations across guidelines meant that many of the initial codes applied across more than one theme. When codes fit across more than one theme, these were assigned to the theme that fit the majority of the codes therein. This flexibility allowed for capture of the nuances of the data set (Naeem et al. 2023; Saunders et al. 2023; Williams and Moser 2019). The first author kept a coding diary to reflect on difficulties, changes, and challenges with the coding process, largely involving the lack of consistency around how guidelines present their recommendations (Berkovic 2023).

## 4 Results

### 4.1 Study Characteristics

The initial search yielded 3344 results. After 1538 duplicates were removed, 1809 titles and abstracts were screened. After applying the inclusion and exclusion criteria, we sought the full text of 373 papers and 342 were excluded based on the full text review (Figure 1) primarily due to the papers not being gray literature/a guideline or recommendation, but rather research articles or reviews. From the initial search, 31 items were included in the full review, with four additional guidelines added from the updated search. Overall, 33 papers were included for review.

The characteristics and the development processes of each guideline are provided in Table 1.

**TABLE 1:** The characteristics and the development processes of each guideline.

									WHO domains addressed?				AACODS domains addressed?					
	Author	Author type	Year	Title	Country/Region	Overall PND or Disease/diagnosis specific?	No. pall care recs	No. total recs	Physical	Social and occupational	Spiritual	Psychosocial	Authority	Accuracy	Coverage	Objectivity	Date	Significance
1	Boostani et al.	Affiliated with government, health service, or university	2023	Iranian clinical practice guideline for amyotrophic lateral sclerosis	Iran	ALS	9	89	Y	Y	N	Y	Y	Y	Y	Y	Y	Y
2	Swiss Medical Weekly	Specific Govt department	2014	Medical‐ethical guidelines: Care and treatment of people with Dementia	Switzerland	Dementia	3	31	Y	Y	Y	Y	Y	Y	Y	Y	Y	Y
3	North West Coast Strategic Clinical Network	Specific Govt department	2018	Palliative Care guidelines in Dementia–2nd Edition	UK	Dementia	4	11	Y	Y	Y	Y	Y	Y	N	Y	Y	Y
4	Palliative Care Australia	Specific Govt department	2014	Palliative Care and Neurological Conditions Position Statement	Australia	General PND	8	8	Y	N	N	Y	Y	Y	N	Y	N	N
5	Rabins et al.	Affiliated with government, health service, or university	2017	Guideline Watch (October 2014): Practice Guideline for the Treatment of Patients with Alzheimer's Disease and Other Dementias	USA	Dementia	2	5	Y	Y	N	Y	Y	N	N	Y	Y	Y
6	Scottish Government	Specific Govt department	2023	Dementia in Scotland: Everyone's Story	Scotland	Dementia	5	10	Y	Y	N	Y	Y	Y	Y	Y	Y	Y
7	Mitchell et al.	Affiliated with government, health service, or university	2016	Palliative and end‐of‐life care for people living with dementia in care homes: part 2	UK	Dementia	7	7	Y	Y	Y	Y	Y	Y	Y	Y	Y	Y
8	Solari et al.	Specific Govt department	2020	EAN Guideline on Palliative Care of People with Severe, Progressive Multiple Sclerosis	Europe	Multiple Sclerosis	8	41	Y	Y	N	Y	Y	Y	Y	Y	Y	Y
9	Grimes et al.	Affiliated with government, health service, or university	2019	Canadian guideline for Parkinson disease	Canada	Parkinson's disease	5	97	Y	N	N	Y	Y	Y	Y	Y	Y	Y
10	Government of Western Australia Department of Health	Specific Govt department	2021	End‐of‐life and Palliative Care for People with Dementia Framework. A framework for developing local palliative care service delivery guidelines for people with dementia	Australia	Dementia	6	6	Y	Y	Y	Y	Y	Y	N	Y	Y	Y
11	Hogarth et al.	Affiliated with government, health service, or university	2017	Consensus clinical management guideline for panthothenate kinase‐associated neurodegeneration (PKAN)	USA & Europe	Panthothenate kinase‐associated neurodegeneration (PKAN)	1	32	Y	Y	Y	Y	Y	Y	Y	Y	Y	Y
12	The Scottish Government	Specific Govt department	2017	Scotland's National Dementia Strategy	Scotland	Dementia	9	36	Y	Y	N	Y	Y	Y	Y	Y	Y	Y
13	National Institute for Health and Care Excellence	Specific Govt department	2019	Motor neurone disease: assessment and management	UK	MND	17	118	Y	Y	N	Y	Y	Y	Y	Y	Y	Y
14	Guideline Adaptation Committee	Specific Govt department	2016	Clinical Practice Guidelines and Principles for Care for People with Dementia	Australia	Dementia	7	109	Y	Y	Y	Y	Y	Y	Y	Y	Y	Y
15	The Irish Palliative Care In Parkinson's Disease Group	Specific Govt department	2016	Palliative care in people with Parkinson's disease. Guidelines for professional healthcare workers on the assessment and management of palliative care needs in Parkinson's disease and related Parkinsonian syndromes	Ireland	Parkinson's disease	30	75	Y	Y	Y	Y	Y	Y	Y	Y	Y	Y
16	National Institute for Health and Care Excellence	Specific Govt department	2017	Parkinson's disease in adults	Ireland	Parkinson's disease	4	100	Y	Y	N	Y	Y	Y	Y	Y	Y	Y
17	Federal Office of Public Health	Specific Govt department	2020	National Dementia Strategy 2014–2019	Switzerland	Dementia	1	19	Y	Y	N	Y	Y	Y	Y	Y	Y	Y
18	Holmerova et al.	Affiliated with government, health service, or university	2016	Dementia Palliare Best Practice Statement	Europe	Dementia	3	20	Y	Y	Y	Y	Y	Y	Y	Y	Y	Y
19	Department of Health	Specific Govt department	2014	The Irish National Dementia Strategy	Ireland	Dementia	5	29	Y	Y	N	Y	Y	Y	Y	Y	Y	Y
20	Shoesmith et al.	Affiliated with government, health service, or university	2020	Canadian best practice recommendations for the management of amyotrophic lateral sclerosis	Canada	ALS	27	131	Y	Y	Y	Y	Y	Y	Y	Y	Y	Y
21	Weafer	Affiliated with government, health service, or university	2014	The palliative care needs of people with advancing neurological disease in Ireland	Ireland	General PND	11	11	Y	Y	N	Y	Y	Y	Y	Y	Y	Y
22	Dementia Clinical Practice Guideline Committee	Specific Govt department	2017	Clinical practice guideline for Dementia 2017	Japan	Dementia	4	119	Y	Y	N	Y	Y	Y	N	Y	Y	Y
23	Marie Curie	Specific Govt department	2015	Triggers for palliative care. Improving access to care for people with diseases other than cancer	UK	General PND	9	9	N	N	N	N	Y	Y	N	Y	Y	N
24	Ministry of Health and Welfare	Specific Govt department	Unknown	The 3rd National Dementia Plan	Korea	Dementia	1	66	Y	Y	N	Y	Y	Y	Y	Y	N	Y
25	National Institute for Health and Care Excellence	Specific Govt department	2018	Dementia: assessment, management and support for people living with dementia and their carers. NICE guideline 97. Methods, evidence and recommendations	UK	Dementia	7	136	Y	Y	Y	Y	Y	Y	Y	Y	Y	Y
26	ALS Society of Canada	Specific Govt department	Unknown	A guide to ALS Patient Care for Primary Care Physicians	Canada	ALS	26	101	Y	Y	Y	Y	Y	Y	Y	Y	Y	Y
27	National Health Service	Specific Govt department	2018	Palliative Care Guidelines in Dementia 2nd Edition	UK	Dementia	4	10	Y	Y	Y	Y	Y	Y	Y	Y	Y	Y
28	Swiss Academy of Medical Sciences	Specific Govt department	2018	Care and treatment of people with dementia	Switzerland	Dementia	15	104	Y	Y	N	Y	Y	Y	Y	Y	Y	Y
29	Dementia Australia	Specific Govt department	2019	Dying Well. Improving Palliative and End of Life Care for people with dementia	Australia	Dementia	11	11	N	Y	N	N	Y	Y	Y	Y	Y	Y
30	Vicic et al.	Affiliated with government, health service, or university	2025	Pan‐Asian consortium for treatment and research in ALS (PACTALS) guidelines for management of amyotrophic lateral sclerosis	Asia Pacific	ALS	17	121	Y	Y	N	Y	Y	Y	Y	Y	Y	Y
31	Tönges et al.	Affiliated with government, health service, or university	2024	Guideline “Parkinson's disease” of the German Society of Neurology (Deutsche Gesellschaft für Neurologie): concepts of care	Germany	Parkinson's disease	10	25	Y	Y	N	Y	Y	Y	Y	Y	Y	Y
32	Urushitani et al.	Affiliated with government, health service, or university	2024	The clinical practice guideline for the management of amyotrophic lateral sclerosis in Japan—update 2023	Japan	ALS	6	106	Y	Y	N	Y	Y	Y	Y	Y	Y	Y
33	Fabrizi et al.	Affiliated with government, health service, or university	2024	The Italian guideline on diagnosis and treatment of dementia and mild cognitive impairment	Italy	Dementia	19	167	Y	Y	N	Y	Y	Y	Y	Y	Y	Y
								Total *N* (%)	32/33 (97)	30/33 (91)	12/33 (36)	31/33 (94)	33/33 (100)	32/33 (97)	27/33 (82)	33/33 (100)	31/33 (94)	31/33 (94)

Guidelines were developed in and for Europe (*n* = 20, 57%), Oceania (*n* = 5, 16%), North America (*n* = 4, 11%), Asia (*n* = 4, 11%), and the Middle East (*n* = 1, 3%). Where more than one continent is represented, it is reported as a “multinational” guideline (*n* = 2, 6%). Most guidelines originated from Europe (UK, Ireland, Scotland, Germany, Italy, and other European nations), and all Oceania guidelines were from Australia (Table S2).

Dementia was the most likely PND to have a specific dedicated guideline (*n* = 18, 51%) (Scottish Government 2017, Dementia Clincial Practice Guideline Development Committee 2017; Department of Health 2014; Fabrizi et al. 2024; Federal Office of Public Health (FOPH) 2019; Government of Western Australia Department of Health 2021; Marie Curie 2015; Guidelide Adaptation Committee 2016; Holmerova et al. 2016; Mitchell et al. 2016; National Institute for Health and Clinical Excellence/Social Care Institute for Excellence 2018; North West Coast Strategic Clinical Network 2018; Rabins et al. 2017; Scottish Government 2023; Swiss Academy of Medical Sciences 2018a).

Other PNDs specifically represented in the guidelines were Multiple Sclerosis (*n* = 1, 3%) (Solari et al. 2020), Amyotrophic Lateral Sclerosis (*n* = 5, 14%) (Amyotrophic Lateral Sclerosis Society of Canada, n.d.; Boostani et al. 2023; National Institute for Health and Care Excellence 2019; Shoesmith et al. 2020; Urushitani et al. 2024; Vucic et al. 2025), Parkinson's disease (*n* = 4, 11%) (Grimes et al. 2019; National Institute for Health and Care Excellence 2017; The Irish Palliative Care in Parkinson's Disease Group 2016; Tönges et al. 2024), and Panthothenate kinase‐associated neurodegeneration (PKAN) (*n* = 1, 3%) (Hogarth et al. 2017). The others were guidelines for general PNDs with no specific condition highlighted (*n* = 4, 11%) (Marie Curie 2015; Palliative Care Australia and Neurological Alliance Australia 2015; Weafer 2014).

The authors of the guidelines were usually official government departments and non‐government disease‐specific agencies (*n* = 18). Other author/funding bodies for guidelines were universities and large groups of authors with multiple university or research affiliations (*n* = 15).

Recommendations totalled 1968 general recommendations and 314 palliative/EOL recommendations.

### 4.2 Quality Assessment

The AACODS scores for each guideline are shown in Table 1. Scores were high across all domains, indicating that the documents are high quality, credible, and appropriate for use in evidence synthesis. Across 33 included documents, AACODS adherence was highest for Authority and Objectivity (both 100%), followed by Accuracy and Significance (97%).

Ninety‐one percent of guidelines (*n* = 31) had a clear date of publication, though in some cases an approximate assumed date was ascertained by additional internet searches.

The lowest score of all AACODS domains was for Coverage, where 71% of the guidelines (*n* = 28) lacked comprehensive scope or did not clearly define the boundaries of their focus (e.g., target population, other exclusions, care setting, or stages of care).

There was high inter‐rater reliability (93%) using the AACODS checklist.

### 4.3 WHO Domains of Palliative Care

None of the papers addressed all four domains of palliative care.

Psychosocial, social, and occupational well‐being were prominently featured across the included papers, with coverage by 91% and 94% of included guidelines respectively. Physical well‐being was also widely considered, with all but one study (97%) incorporating this domain (Marie Curie 2015). In contrast, spiritual wellbeing was the least frequently addressed domain, examined in only 12 of the 34 studies (36%).

One paper did not directly address any of the domains but was an evidence‐based call to action for increased awareness of what palliative care needs were present for different diseases, and the professional skills required to provide high‐quality and holistic care (Marie Curie 2015).

### 4.4 Themes

Inductive content analysis generated a set of categories that captured the key patterns and concepts evident across the dataset. Six overarching themes reflected best practice in person‐centered palliative care for people living with PND, their families, and the teams coordinating care. Collectively, these themes underscore the central importance of delivering holistic, person‐centered palliative care in PND. These themes are not discrete; rather, they are interwoven and mutually reinforcing, collectively shaping a comprehensive, holistic approach to person‐centered palliative care in PND. The themes are:

1. Holistic and compassionate symptom management is essential in PND across the PND trajectory.
2. Recognizing families and caregivers as integral care partners with their own distinct care needs.
3. The need for early, frequent, and dynamic communication and conversations across the disease course.
4. Enabling meaningful choice‐making aligned with patient values.
5. The multidisciplinary team (MDT) expertise is the foundation for high‐quality care; and
6. Navigating medicolegal complexities.

The six themes are described in detail below, with exemplars from the guidelines to illustrate these themes.

#### 4.4.1 Theme 1. Holistic and Compassionate Symptom Management Is Essential in PND Across the PND Trajectory

Across all included guidelines, holistic and compassionate symptom management was identified as a core component of best‐practice palliative care for people living with PND. This includes physical, psychological, psychosocial, and spiritual symptoms. Guidelines consistently emphasized that symptom management should be integrated throughout the disease trajectory, not just at the EOL. Symptom management was described as dynamic and non‐linear, shaped by diagnosis, disease progression, and the individual's preferences, priorities, and goals. Consequently, symptom management was framed as an iterative and cyclical process requiring ongoing assessment, review, and adaptation.

As disease advances and functional decline increases, guidelines noted that symptom management often becomes more complex, particularly in the context of multimorbidity, cognitive impairment, and increased care dependence. While physical symptoms remain central, high‐quality palliative care was described as extending beyond physical management alone to encompass psychological, social, and spiritual dimensions of care, alongside explicit consideration of family and caregiver needs. This holistic, person‐centered approach was emphasized across guidelines, with care plans expected to integrate symptom control with psychosocial and spiritual support, delivered with compassion and coordinated across care teams:

An individual plan of care, which includes food and drink, symptom control, and psychological, social and spiritual support, is agreed, coordinated and delivered with compassion. (North West Coast Strategic Clinical Network 2018)

Several guidelines explicitly cautioned against equating high‐quality palliative care with physical symptom control alone. Instead, holistic care was positioned as inseparable from person‐centeredness, requiring attention to emotional distress, anxiety, depression, existential concerns, and the relational impacts of PND:

The physical care of a person who is dying is essential. However, there should also be a consistent focus on the psychological, social and spiritual needs of the person by providing holistic care. The ethos of holistic care corresponds directly with the theory of person‐centred care […]. People living with dementia may become anxious, distressed or depressed during the remainder of their life […]. Providing of person‐centred care can be beneficial; talking to the person, using touch, providing meaningful activity and reminiscence therapy should be embedded in care home practice […]. Effective communication enables these examples of best practice to be used in improving care […]. (Mitchell et al. 2016)

As individuals approach the EOL, guidelines highlighted the importance of proactively addressing decisions related to the continuation, withholding, or withdrawal of life‐sustaining treatments, including medications, artificial nutrition and hydration, ventilation, and cardiopulmonary resuscitation. Early and ongoing discussions with individuals and families were emphasized to support informed decision‐making and allow sufficient time to consider preferences and values:

The focus [in severe dementia] increasingly shifts towards EOL issues (management of nutrition and hydration, possible withholding of life‐extending measures, accompaniment of the dying patient). (Swiss Academy of Medical Sciences (SAMS) 2018a)

Overall, this theme demonstrates that guidelines conceptualize symptom management in PND as a holistic, compassionate, and continuous process that, integrating physical, psychological, social, and spiritual dimensions in response to evolving needs across the illness trajectory.

#### 4.4.2 Theme 2. Recognizing Families and Caregivers as Integral Care Partners With Their Own Distinct Care Needs

Across all included guidelines, families and informal caregivers were consistently positioned as central to the care of people living with PND. Their role was described as extending beyond practical support to encompass advocacy, decision‐making, and the emotional responsibilities of sustaining relationships throughout progressive decline. Guidelines emphasized the importance of recognizing family members' experiential knowledge, actively valuing their expertise, and integrating their perspectives into care planning and clinical decision‐making.

Families and caregivers were not only framed as supporters of the person with PND but also as individuals with their own care needs. Several guidelines explicitly characterized families and informal caregivers as “patients” in their own right, with physical, psychological, social, and spiritual needs that require ongoing assessment and support. Inclusion of families in discussions, reviews, and treatment planning was consistently recommended, wherever possible, as part of person‐centred and holistic care.

The importance of continuing care for families beyond the death of the person with PND was also strongly emphasized. Bereavement support was described as a core component of high‐quality palliative and EOL care, rather than an optional or time‐limited add‐on. Guidelines highlighted the diversity of grief experiences and the need for flexible, individualized approaches to bereavement support:

The family plays an important role in end‐of‐life care. At the same time, since the family is under great mental and physical stress, support for the family is important. In addition, after the person with dementia has died, grief care for the family is necessary. (Dementia Clinical Practice Guideline Development Committee 2017)

Caregiver burden and burnout were identified as significant and common risks across the disease trajectory. Guidelines highlighted that ensuring the care needs of the person with PND are adequately met by the healthcare team can help mitigate caregiver strain by enabling family members to maintain their social and relational roles, rather than assuming increasingly complex practical or medical responsibilities. Strong collaboration with community‐based services was identified as critical to maintaining caregiver wellbeing, particularly during periods of change and stress such as transition or escalation in care needs.

Carers are at an increased risk of poor health and their needs should be assessed and reviewed regularly by their own health practitioner. Carer and family needs should be addressed regularly, including if the person with dementia has entered residential care, and after their death. (Guidelide Adaptation Committee 2016)

Guidelines also highlighted the emotional and ethical complexity faced by families when involved in decisions about life‐sustaining treatments, such as enteral feeding, ventilation, or tracheostomy. These decisions were noted to be particularly challenging when families perceived such actions as potentially hastening death or causing suffering. As such, guidelines stressed the importance of clear, timely, and compassionate communication about disease progression, end‐stage care, and the dying process. While acknowledging clinicians' concerns about the emotional impact of these discussions, several guidelines cautioned that withholding or delaying information may unintentionally increase distress and contribute to poorly informed decision‐making, thereby negatively affecting caregiver wellbeing. The need for proactive assessment of bereavement risk and tailored post‐death support was reinforced:

NICE highlights that bereavement can cause a wide range of needs, including practical, financial, social, emotional, and spiritual. The assessment of a carer's bereavement risks should enable an appropriate plan to be put in place to meet their identified needs. […] Access to additional support should be offered to those for whom this is insufficient or in whom it is anticipated that difficult grief reactions may be experienced. (North West Coast Strategic Clinical Network 2018)

Overall, this theme underscores that guidelines conceptualize families and caregivers as indispensable partners in PND care, whose needs span the physical, emotional, social, and spiritual domains and persist beyond the death of the person with PND. Effective palliative care for PND therefore requires systematic inclusion, assessment, and ongoing support of families and caregivers across the entire care trajectory.

#### 4.4.3 Theme 3. The Need for Early, Frequent, and Dynamic Communication and Conversations Across the Disease Course

Analysis of the included guidelines consistently identified early, frequent, and dynamic communication as essential to high‐quality palliative and EOL care for people living with PND. There is no universally agreed understanding of what constitutes an “early” discussion of palliative care and future planning; however, Mitchell et al. (2016) assert that palliative care starts at the time of diagnosis. Guidelines emphasized that timely and ongoing conversations support shared understanding, preparedness, and responsive care, particularly as the disease progresses and needs evolve. Regular review of palliative and EOL care needs was recommended to account for changes in physical function, cognition, symptom burden, and personal preferences.

Throughout the guidelines, communication was conceptualized as an iterative process that should adapt over time, rather than a single planning event. Thematic patterns highlighted that individuals' wishes, priorities, and goals are likely to change as PND progresses, and that decision‐making capacity may become impaired or fluctuate. As such, revisiting and updating palliative and EOL plans was viewed as integral to person‐centered care. While multidisciplinary teams and families were identified as key participants in these discussions, the person living with PND was consistently positioned as central, even when decision‐making capacity is limited or declining. Early future planning was strongly emphasized to ensure that individuals have the opportunity to articulate their values and preferences while they have sufficient capacity to do so. However, guidelines also acknowledged the variability in timing and circumstances of diagnosis, reinforcing the need for flexibility and responsiveness in planning processes:

It is recommended that planning for the future takes place at an early stage so that the person with dementia has sufficient cognitive abilities to articulate goals and desired direction of care. However, diagnosis, can happen at different points in the illness and sometimes not at all; future planning will need to reflect the differing circumstances so the person can live the best life possible and prepare for potentially challenging times ahead. (Holmerova et al. 2016)

Advance care planning and advance care directives emerged as central mechanisms through which ongoing communication could be structured and documented. Guidelines consistently recommended that conversations about advance care planning begin early and be embedded within routine follow‐up and review processes. Inclusion of families and caregivers in these discussions was emphasized, alongside clear documentation of preferences at each stage. Decisions regarding the withholding or withdrawal of life‐sustaining treatments (such as cardiopulmonary resuscitation, medications, or other medical interventions) were identified as important components of advance care planning, subject to legal and jurisdictional requirements:

[…] those with fluctuating capacity, are given the opportunity to discuss advance care planning at each health and social care review. (Fabrizi et al. 2024)

As part of a broader holistic planning approach, guidelines highlighted the importance of exploring individuals' values, understanding of their illness, and spiritual or existential concerns. When decision‐making capacity was lost, medical professionals were encouraged to work collaboratively with trusted family members or carers, guided by previously expressed wishes and formal documentation. Regular, formal assessment of decision‐making capacity was recommended to support ethical and legally sound decision‐making.

It is recommended to seek the patient's opinion about important foreseeable future decisions, such as tracheostomy and life‐sustaining measures before they lose their decision‐making capacity. Otherwise, the patient should be guided to choose their surrogate in advance directive. (Boostani et al. 2023)

Overall, this theme highlights that guidelines position communication as a continuous, relational, and adaptive process that underpins effective palliative and EOL care in PND. Early initiation, regular review, and responsiveness to change were consistently emphasized as critical to supporting the communication necessary for informed, value‐congruent decision‐making across the disease trajectory.

#### 4.4.4 Theme 4. Enabling Meaningful Choice‐Making Aligned With Patient Values

The included guidelines identified enabling meaningful choice‐making aligned with individual values as a central principle of person‐centred palliative and EOL care for people living with PND. Guidelines consistently described the need for processes that facilitate informed and value‐congruent decision making (such as the communication and conversation processes described in Theme 3). Theme 4 captures the focus on empowering individuals with PND to exercise autonomy in care choices despite complex and evolving circumstances.

Guidelines were consistent in emphasizing that informed consent is the cornerstone of ethical and person‐centered care. A person should be equipped with all available tools and information to make informed and evidence‐based choices about their care. Enabling choice and control over care includes providing accessible information and ensuring access to care is fair and equitable regardless of diagnosis, socioeconomic background, or other factors. Several guidelines highlighted the challenges posed by jurisdictional variation in legislation and policy, particularly in relation to advance care planning and advance care directives. Findings underscored calls for greater consistency and clarity to support informed decision‐making and continuity of care across settings, and ensuring equality and equity of access to care:

Start the dialogue about consistent advanced care planning legislation to reduce jurisdictional confusion, provide protection to health professionals and community members, and allow care recipients to transition across borders to be closer to family and their community. (Dementia Australia 2019)

People with Parkinson's disease and their family members and caregivers should be given appropriate verbal and written information about the following, and it should be recorded that the discussion has taken place (progression of Parkinson's disease, effects of medication, advanced care planning and treatment orders ETC), options for future management, what could happen at the EOL, available support services. (Grimes et al. 2019)

While cognitive impairment is not always a barrier to effective choice‐making, guidelines consistently noted that impairment should not be assumed to preclude meaningful participation. Early advance care planning discussions ensures the person with PND can fully participate prior to any decline in decision‐making capacity. Planning for ongoing treatment and EOL may even start at the time of diagnosis so the person can contribute fully. Where individuals were no longer able to express their wishes, decision‐making based on best‐interest principles, informed by previously documented preferences and values, was recommended.

Palliative care should be accessible, fair, equitable, and provided to any person or family member who wants it, in the location(s) they choose to access it, and in a way that is culturally sensitive. This includes preferences around place of death. Information should be presented in an accessible format, which may be written or verbal.

People receive health and social care that supports their wellbeing, irrespective of diagnosis, age, socioeconomic background, care setting or proximity to death. (Marie Curie 2015)

Analysis also highlighted a strong emphasis on values‐based equity in access to palliative care. Guidelines consistently framed fair and equitable access as an ethical imperative, emphasizing that palliative care should be available to all individuals and families, regardless of diagnosis, care setting, or circumstances. This reflects a broader principle that equity of access is inseparable from meaningful choice‐making, since choice is only meaningful if the options presented are genuinely available to the person. Where systemic inequities prevent this, a person's choice becomes conditional on factors that should be irrelevant. Access to care was further linked to respect for individual values and preferences, including where care is delivered and preferences regarding place of death:

Palliative care should be available to all people with neurological conditions who need and want it. (Palliative Care Australia and Neurological Alliance Australia 2015)

Overall, this theme demonstrates that guidelines consistently recommend and conceptualize choice‐making as a values‐driven, relational, and supported process. Enabling this meaningful choice in PND care requires early and ongoing communication, equitable access to services, clear legal and policy frameworks, and systems that support individuals to make informed decisions aligned with their values across the disease trajectory.

#### 4.4.5 Theme 5: The Multidisciplinary Team Expertise Is the Foundation for High‐Quality Care

Analysis of the guidelines consistently identified the expertise of the multidisciplinary team (MDT) as foundational to the delivery of high‐quality palliative and EOL care for people living with PND. Guidelines conceptualized the MDT as a coordinated group of skilled health and care professionals responsible for addressing the complex and evolving needs of both individuals with PND and their families. Across documents, the quality, confidence, and consistency of care were directly linked to the collective knowledge, skills, and training of the team.

Guidelines described MDTs as skilled professionals typically comprising medical practitioners (including neurologists and palliative care specialists), nurses, paid care workers, and allied health professionals, each contributing distinct forms of expertise. Specialist competency in palliative care was consistently emphasized as essential across roles, with guidelines underscoring the need for ongoing education and training in symptom management, communication, advance care planning, ethical decision‐making, and EOL care (Dementia Australia 2019; Marie Curie 2015; The Ministry of Health and Welfare 2016; Weafer 2014). Rather than positioning expertise as static, guidelines framed competency development as an ongoing process requiring organizational support and access to continuing professional development opportunities.

Increase workforce training about the unique palliation needs of people with dementia to aged care workers, GPs, and acute care staff. (Dementia Australia 2019)

In addition to clinical skills, guidelines highlighted the importance of professionals understanding their medicolegal and ethical responsibilities. Policies and procedures were described as playing a key role in enabling safe and effective practice by supporting training, scope‐of‐practice clarity, and timely referral or consultation. A strong emphasis was placed on professional humility and collaboration, with guidance recommending that team members recognize the limits of their own expertise and actively seek input from other disciplines when required:

Care teams should be multidisciplinary and care professionals should acknowledge when their own experience is limited and when to contact other specialists for support. (The Irish Palliative Care in Parkinson's Disease Group 2016)

Given the long‐term and often close relationships that develop between MDT members, patients, and families over the course of PND, guidelines also highlighted the emotional responsibilities and labour inherent in this work. Analysis identified staff wellbeing as a recurring concern, with recommendations that MDTs have access to psychosocial supports to mitigate stress, moral distress, and burnout:

Support for staff should include peer support, informal debriefing sessions, or counselling as appropriate. (The Irish Palliative Care in Parkinson's Disease Group 2016)

At the organizational level, guidelines emphasized the responsibility of health services and internal policy makers to ensure MDTs are adequately resourced. This included access to workforce training, palliative care competencies, clear clinical guidelines, and the practical resources required to deliver care aligned with identified needs. Insufficient resources were described as contributing to professional stress and undermining confidence in care delivery:

basic resources to provide the level of care that they feel the person needs [in order to] reduce the professional stress when there is a need that they can't address. (The Irish Palliative Care in Parkinson's Disease Group 2016)

Only one guideline (Vucic et al. 2025) took into careful consideration the cultural and socioeconomic differences in availability and acceptability of palliative and EOL care. The authors describe the impact of this on availability of specialist skills and knowledge in certain geographical areas.

In settings where palliative care is not yet developed, the healthcare worker closely linked to the patient is best positioned to deliver this care and as such should be given access to necessary training (Vucic et al. 2025)

Overall, this theme highlights that guidelines position MDT expertise not merely as a desirable feature but as a prerequisite for high‐quality, equitable, and sustainable palliative care in PND. Equitable care requires not just access to a service, but access to the same breadth of expertise regardless of setting. This depends on clinicians across disciplines being appropriately skilled, which in turn requires policy and governance support. MDT expertise a precondition for, rather than incidental to, equitable care. Effective care was consistently linked to coordinated multidisciplinary working, ongoing competency development, organizational support, and attention to the wellbeing of the workforce delivering care.

#### 4.4.6 Theme 6. Navigating Medicolegal Complexities

Guidelines frequently identified medicolegal considerations as a pervasive and influential factor shaping palliative and EOL care for people living with PND. They highlighted the influence of medicolegal considerations on care processes, including the ongoing tensions, uncertainties, and responsibilities clinicians must balance when working within legal and ethical boundaries. Adding further complexity is that the medicolegal requirements of providing and accessing palliative/EOL care can differ between and within countries, states, and regions.

Advance care directives and advance care planning were repeatedly emphasized as critical tools for supporting legally and ethically sound care. Guidelines framed the early initiation and regular review of directives as essential to ensuring that individuals' wishes and values remain central to care decisions, as well as reducing caregiver burden by limiting the difficult decisions that families are often required to make particularly when acting as formal substitute decision‐makers.

Considerable attention was given to legally sanctioned practices related to hastening death, including voluntary assisted dying (VAD), physician‐assisted suicide (PAS), and other forms of medically assisted dying. Guidelines acknowledged that the legality and eligibility criteria for these practices vary substantially by country, jurisdiction, and diagnosis. Any request for medically assisted dying was framed as requiring careful handling by the treating clinician and strict adherence to local legislation and professional guidance. In some contexts, medically assisted dying was described as a possible option alongside other forms of symptom management, subject to legal requirements and rigorous clinical assessment (Shoesmith et al. 2020).

Across guidelines, the complexity of desires to hasten death was strongly emphasized. Rather than treating such wishes as singular or straightforward, documents highlighted the need for specialist knowledge and training to differentiate between a sustained, legally relevant request and expressions of distress rooted in potentially modifiable factors such as depression, social isolation, loss of function, or fear of burdening others:

Healthcare professionals should be aware of the risk factors for the wish for hastened death – including depression, isolation, restricted abilities – and encourage the discussion of these issues and the appropriate management. (Solari et al. 2020)

Guidelines also drew attention to the risk of suicide that is not medically assisted. This risk was often linked to distress following diagnosis, fear of future dependence, perceived loss of identity, and concerns about becoming a burden. Several guidelines emphasized the importance of empathetic disclosure of diagnosis, ongoing psychosocial support, and early identification and treatment of mental health conditions as protective factors. Even in jurisdictions where assisted suicide is legally permissible under specific conditions, guidelines stressed that assessing decision‐making capacity in the context of cognitive decline is particularly challenging and demands a high level of clinical expertise, ethical sensitivity, and procedural rigor.

People diagnosed with dementia may […] express a wish to end their own life, often to avoid complete dependence or becoming a burden. Empathetic disclosure of the diagnosis, along with continued support and information, can help diminish such fears. Any emotional or mental health problems contributing to suicidal desire must be identified and treated. If the desire persists, assisted suicide is legally permissible for patients with capacity, though assessing capacity in dementia is particularly demanding. (Swiss Academy of Medical Sciences 2018b)

Overall, this theme highlights that guidelines conceptualize medicolegal considerations as deeply interwoven with clinical, ethical, and relational aspects of PND care. Navigating these complexities requires early and ongoing planning, clear documentation, regular capacity assessment, specialist knowledge, and strong communication with individuals and families. High‐quality palliative care in PND is therefore dependent not only on clinical skill but also on clinicians' ability to work confidently and compassionately within complex medicolegal environments.

## 5 Discussion

In the context of substantial international variation in how palliative care is defined, delivered, and standardized, this review provides the first systematic mapping of guidelines, protocols, and healthcare standards for palliative care in PNDs. Using a comprehensive search strategy spanning academic databases, gray literature, and targeted web searches, 35 relevant guidelines were identified. Together, these documents demonstrate the breadth of existing considerations of contemporary international guidance while also revealing notable gaps in scope, consistency, and comprehensiveness.

Inductive content analysis revealed six higher‐order themes reflecting the dynamic, interconnected nature of palliative care in PND. Together, these themes reinforce holistic, person‐centered care that addresses physical, psychosocial, and existential needs. Families and informal caregivers emerged as integral care partners, underscoring the need to actively address caregiver burden, recognizing the caregiver's role as integral to sustaining quality of life for people with PND while also acknowledging caregivers as having their own holistic support needs as they navigate role changes, anticipatory grief, and symptoms of caregiver burden. Early palliative care integration, proactive communication, and advance care planning were consistently emphasized across guidelines as essential to value‐aligned decision‐making. Future investigations could define what “early”, while remaining person‐centered and disease‐specific. Consensus on a definition of “early” will benefit policy‐makers, provide guidance and supports for care teams, and may help patients and families in setting expectations for disease trajectories. Well‐coordinated, adequately trained MDTs and clarity around medicolegal and ethical considerations were similarly underscored. Although derived inductively, these themes align closely with the WHO domains of palliative care.

These guidelines were predominantly developed in high‐income regions, particularly Europe and Australia, with comparatively limited representation from Asia and the Middle East and a small proportion identified as global guidelines. Most guidelines were authored or funded by government bodies and non‐government, disease‐specific organizations, underscoring the central role of policy and advocacy groups in shaping palliative care practice in this field. Dementia emerged as the condition most frequently supported by dedicated guidance, accounting for almost half of the included documents, while other PNDs associated with specific diseases (such as Amyotrophic Lateral Sclerosis, Parkinson's disease, multiple sclerosis, and rarer conditions) were far less consistently addressed, highlighting an imbalance in condition‐specific guidance. This imbalance likely reflects differences in disease prevalence, advocacy strength, and funding priorities, but it also raises concerns about equity in access to condition‐specific guidance. Given the distinct clinical trajectories, symptom profiles, and psychosocial impacts associated with different PNDs, reliance on broad or dementia‐focused guidance risks obscuring condition‐specific needs and may limit the relevance of recommendations for some populations.

Encouragingly, the methodological quality of the included guidelines was high overall. Using the AACODS checklist, most documents demonstrated strong performance across domains of authority, objectivity, and accuracy, with high inter‐rater reliability supporting the robustness of the appraisal process. However, despite high overall quality, gaps in coverage were common. Many guidelines did not clearly define their scope, target populations, care settings, or stages of disease, creating potential ambiguity for implementation in clinical practice.

Alignment with WHO palliative care domains was variable: while physical, psychosocial, and social domains were widely addressed, spiritual wellbeing was notably underrepresented, with only one‐third of guidelines comprehensively incorporating it. This gap mirrors broader challenges in operationalizing spiritual care within health systems but is particularly salient in PND, where existential distress, meaning‐making, and fears about dependency, identity, and dying are often prominent (Bolt et al. 2025; Ćurković et al. 2026; Husic et al. 2025). Future guideline development would benefit from more explicit integration of spiritual care, including practical guidance for clinicians, referral pathways, and culturally sensitive approaches.

Across the articles we located, we identified 1968 general care recommendations and 314 palliative/EOL recommendations. Although fewer recommendations were explicitly labeled as palliative or EOL care, this should not be interpreted as a lack of palliative focus. Rather, many guidelines embedded palliative principles throughout the disease course, integrating symptom management, psychosocial support, and anticipatory planning well before the terminal phase.

Several limitations should be acknowledged. While the AACODS checklist supported a structured and transparent quality appraisal and has been used extensively locally and internationally (Bandyopadhyay et al. 2020; Coward et al. 2025; Jatau et al. 2021; Kennedy et al. 2021; Khosravi et al. 2025; Xiong et al. 2024), some criteria rely on subjective judgment and assume substantial domain expertise, which may introduce bias despite strong inter‐rater agreement. For example, the ‘Authority’ criterion asks “[does the author have] professional qualifications or considerable experience?” and the ‘Significance’ criterion asks whether the publication is meaningful, adds contexts, enriches or adds something unique to the research, strengthens or refutes a current position, whether it has impact, whether the research area would be lesser without it, whether it is integral/representative/typical, and whether it has impact. Additionally, some of the checklist items assume a high level of knowledge and experience with the subject matter. For example, “does it have impact?” and “is it representative of work in the field?” The checklist has been modified by the National Institute for Clinical and Health Excellence (NICE) in the United Kingdom to include “partly” and “unclear” as additional descriptors but still leaves the decision open to interpretation (National Institute for Health and Care Excellence (NICE) 2014).

The predominance of guidelines from high‐income regions limits the generalizability of findings to low‐ and middle‐income contexts, where resource constraints and service structures differ substantially. Using the Country Classification from the World Bank (The World Bank 2025) to determine a country's economic status, all but two of the 33 papers originate from high‐income countries or regions. The only representation from low‐ or middle‐income countries is from the Asia Pacific region (Vucic et al. 2025). Additionally, variability in how recommendations were presented (ranging from clearly delineated lists to embedded narrative statements) made consistent identification and synthesis challenging. Future reviews could strengthen methodological rigor by including dual independent data extraction on the full set of included papers and adopting more standardized approaches to defining and categorizing recommendations.

## 6 Conclusion

This review demonstrates that while existing palliative care guidelines for PND are generally high quality and internationally influential, they are unevenly distributed across conditions and regions and vary in their comprehensiveness. Addressing gaps in spiritual care, caregiver support, condition‐specific guidance, and global representation represents a critical opportunity to inform future guideline development, policy formulation, and service planning. Greater harmonization of scope and clearer articulation of palliative principles across the disease trajectory may enhance the consistency, equity, and person‐centredness of palliative care for people living with PND and their families worldwide.

This review also has direct implications for both clinical practice and policy. By synthesizing core recommendations from high‐quality guidelines, the findings provide a credible evidence base to inform clinical decision‐making, health service design, and policy development aimed at strengthening palliative and EOL care for people with PND, irrespective of diagnosis or geographical location. In addition, the review offers patients and carers a consolidated reference that may support self‐advocacy for best‐practice care as they navigate complex healthcare systems.

## 7 Clinical Resources

World Health Organization (WHO)—Palliative Care: Palliative care.

International Association for Hospice and Palliative Care: International Association for Hospice & Palliative Care.

Worldwide Hospice and Palliative Care Alliance: The Worldwide Hospice Palliative Care Alliance.

## Funding

This research was supported by the Commonwealth through an Australian Government Research Training Program Scholarship. https://doi.org/10.82133/C42F‐K220.

## Conflicts of Interest

The authors declare no conflicts of interest.

## Supporting information


**Table S1:** Search terms.
**Table S2:** Characteristics of the guidelines.
**Table S3:** AACODS checklist.

## Data Availability

All data generated or analyzed during this study are included in this published article and the Supporting Information are available upon reasonable request from the corresponding author. This research was supported by the Commonwealth through an Australian Government Research Training Program Scholarship (https://doi.org/10.82133/C42F‐K220).

## References

[jnu70130-bib-0002] Amyotrophic Lateral Sclerosis Society of Canada . n.d. “A Guide to ALS Patient Care For Primary Care Physicians.” https://als.ca/wp‐content/uploads/2021/05/A‐Guide‐to‐ALS‐Patient‐Care‐For‐Primary‐Care‐Physicians‐English.pdf.

[jnu70130-bib-0003] Aubeeluck, A. 2005. “Caring for the Carers: Quality of Life in Huntington's Disease.” British Journal of Nursing 14, no. 8: 452–454. 10.12968/bjon.2005.14.8.17929. 15924027

[jnu70130-bib-0004] Bandyopadhyay, S. , R. E. Baticulon , M. Kadhum , et al. 2020. “Infection and Mortality of Healthcare Workers Worldwide From COVID‐19: A Systematic Review.” BMJ Global Health 5, no. 12: e003097. 10.1136/bmjgh-2020-003097. PMC7722361 33277297

[jnu70130-bib-0005] Berkovic, D. 2023. “Chapter 21: Content Analysis.” In Qualitative Research—A Practical Guide for Health and Social Care Researchers and Practitioners. Monash University Library. https://oercollective.caul.edu.au/qualitative‐research/chapter/__unknown__‐21/.

[jnu70130-bib-0006] BISA . 2025. Progressive Neurological Disorders. Brain Injury SA (BISA). https://braininjurysa.org.au/progressive‐neurological‐disorders/.

[jnu70130-bib-0007] Boersema‐Wijma, D. J. , E. van Duijn , A.‐W. Heemskerk , J. T. van der Steen , and W. P. Achterberg . 2023. “Palliative Care in Advanced Huntington's Disease: A Scoping Review.” BMC Palliative Care 22, no. 1: 54. 10.1186/s12904-023-01171-y. 37138329 PMC10155365

[jnu70130-bib-0008] Bolt, S. , S. Metselaar , T. Versteeg , and C. Kröger . 2025. “Addressing Spirituality‐Related Moral Challenges in Palliative Care: Perspectives of Spiritual Counselors.” Journal of Health Care Chaplaincy 31, no. 4: 292–312. 10.1080/08854726.2025.2471740. 40029334

[jnu70130-bib-0009] Boostani, R. , N. Olfati , H. Shamshiri , et al. 2023. “Iranian Clinical Practice Guideline for Amyotrophic Lateral Sclerosis.” Frontiers in Neurology 14: 1154579. 10.3389/fneur.2023.1154579. 37333000 PMC10272856

[jnu70130-bib-0010] Bos, I. , K. Wynia , J. Almansa , G. Drost , B. Kremer , and J. Kuks . 2019. “The Prevalence and Severity of Disease‐Related Disabilities and Their Impact on Quality of Life in Neuromuscular Diseases.” Disability and Rehabilitation 41, no. 14: 1676–1681. 10.1080/09638288.2018.1446188. 29514523

[jnu70130-bib-0011] Braun, V. , and V. Clarke . 2022. Thematic Analysis: A Practical Guide. Sage.

[jnu70130-bib-0012] Chiao, C.‐Y. , H.‐S. Wu , and C.‐Y. Hsiao . 2015. “Caregiver Burden for Informal Caregivers of Patients With Dementia: A Systematic Review.” International Nursing Review 62, no. 3: 340–350. 10.1111/inr.12194. 26058542

[jnu70130-bib-0013] Clark, S. E. , G. Chisnall , and C. Vindrola‐Padros . 2022. “A Systematic Review of de‐Escalation Strategies for Redeployed Staff and Repurposed Facilities in COVID‐19 Intensive Care Units (ICUs) During the Pandemic.” EClinicalMedicine 44: 101286. 10.1016/j.eclinm.2022.101286. 35156007 PMC8820730

[jnu70130-bib-0014] Coward, C. , J. Hunter , S. M. Halliday , B. Jeffers , and D. Hopkins‐Rosseel . 2025. “Entry‐to‐Practice Business and Practice Management Competencies: A Qualitative Systematic Review to Inform Canadian Physiotherapy Curricula.” Physiotherapy Canada 77, no. 1: 69–78. 10.3138/ptc-2022-0051. 40959722 PMC12392823

[jnu70130-bib-0015] Curie, M. 2015. Triggers for Palliative Care. Improving Access to Care for People With Diseases Other Than Cancer. Marie Curie.

[jnu70130-bib-0016] Ćurković, A. , M. Dolić , and L. Lušić Kalcina . 2026. “Nurses' Perspectives on Unmet Social, Psychological, and Spiritual Needs of Palliative Patients in Croatia: A Cross‐Sectional Study.” Nursing Reports 16, no. 1: 29. 10.3390/nursrep16010029. 41591125 PMC12844772

[jnu70130-bib-0017] Decruyenaere, M. , G. Evers‐Kiebooms , A. Boogaerts , K. Demyttenaere , R. Dom , and J.‐P. Fryns . 2005. “Partners of Mutation‐Carriers for Huntington's Disease: Forgotten Persons?” European Journal of Human Genetics 13, no. 9: 1077–1085. 10.1038/sj.ejhg.5201462. 15999117

[jnu70130-bib-0018] Dementia Australia . 2019. Dying Well Improving Palliative and End of Life Care for People With Dementia. Dementia Australia. https://www.dementia.org.au/sites/default/files/documents/DA%20Dying%20Well%20Discussion%20Paper%20FINAL%20for%20online.pdf.

[jnu70130-bib-0019] Dementia Clincial Practice Guideline Development Committee . 2017. “Clinical Practice Guideline for Dementia 2017.” https://neurology‐jp.org/guidelinem/dementia/documents/guideline2017.pdf.

[jnu70130-bib-0020] Department of Health . 2014. The Irish National Dementia Strategy. Department of Health. https://assets.gov.ie/10870/3276adf5273f4a9aa67e7f3a970d9cb1.pdf.

[jnu70130-bib-0021] Durepos, P. , A. Wickson‐Griffiths , A. A. Hazzan , et al. 2017. “Assessing Palliative Care Content in Dementia Care Guidelines: A Systematic Review.” Journal of Pain and Symptom Management 53, no. 4: 804–813. 10.1016/j.jpainsymman.2016.10.368. 28063859

[jnu70130-bib-0022] Elamri, N. , I. Atif , A. Lyazidi , M. Rattal , and A. Gantare . 2025. “Impact of Progressive Neurological Disorders on Patients' Daily Lives.” Journal of Evidence‐Based Care 15, no. 2: 54–61. 10.22038/EBCJ.2025.86813.3109.

[jnu70130-bib-0023] Fabrizi, E. , A. Ancidoni , N. Locuratolo , et al. 2024. “The Italian Guideline on Diagnosis and Treatment of Dementia and Mild Cognitive Impairment.” Age and Ageing 53, no. 11: afae250. 10.1093/ageing/afae250. 39544104 PMC11564805

[jnu70130-bib-0024] Federal Office of Public Health (FOPH) . 2019. National Dementia Strategy 2014–2019. FOPH and CMPH.

[jnu70130-bib-0025] Government of Western Australia Department of Health . 2021. End‐of‐Life and Palliative Care for People With Dementia Framework. A Framework for Developing Local Palliative Care Service Delivery Guidelines for People With Dementia. Department of Health.

[jnu70130-bib-0026] Grimes, D. , M. Fitzpatrick , J. Gordon , et al. 2019. “Canadian Guideline for Parkinson Disease.” Canadian Medical Association Journal 191, no. 36: E989–E1004. 10.1503/cmaj.181504. 31501181 PMC6733687

[jnu70130-bib-0027] Guidelide Adaptation Committee . 2016. Clinical Practice Guidelines and Principles for Care for People With Dementia. Guideline Adaptation Committee. https://cdpc.sydney.edu.au/wp‐content/uploads/2019/06/CDPC‐Dementia‐Guidelines_WEB.pdf.

[jnu70130-bib-0028] Hogarth, P. , M. A. Kurian , A. Gregory , et al. 2017. “Consensus Clinical Management Guideline for Pantothenate Kinase‐Associated Neurodegeneration (PKAN).” Molecular Genetics and Metabolism 120, no. 3: 278–287. 10.1016/j.ymgme.2016.11.004. 28034613

[jnu70130-bib-0029] Holmerova, I. , A. Waugh , R. MacRae , et al. 2016. Dementia Palliare Best Practice Statement. University of the West of Scotland. https://www.uws.ac.uk/palliareproject.

[jnu70130-bib-0030] Husic, S. , B. Miletic , T. Stemberger Kolnik , and V. Vejzovic . 2025. “Emotional and Spiritual Challenges of Informal Caregivers: The Need for Early Mobile Palliative Care Support.” Nursing Reports (Pavia, Italy) 15, no. 9: 327. 10.3390/nursrep15090327. 41003282 PMC12472654

[jnu70130-bib-0031] Jatau, A. I. , A. Sha'aban , K. A. Gulma , et al. 2021. “The Burden of Drug Abuse in Nigeria: A Scoping Review of Epidemiological Studies and Drug Laws.” Public Health Reviews 42: 1603960. 10.3389/phrs.2021.1603960. 33796340 PMC7904248

[jnu70130-bib-0032] Kennedy, K. J. , D. Forsythe , J. Wagner , and M. Eckert . 2021. “Clinical Pathways for the Evidence‐Based Management of Behavioural and Psychological Symptoms of Dementia in a Residential Aged Care Facility: A Rapid Review.” Australasian Journal on Ageing 40, no. 4: 347–355. 10.1111/ajag.12990. 34342112

[jnu70130-bib-0033] Khosravi, M. , S. M. Mojtabaeian , and Z. Zare . 2025. “Factors Influencing the Use of Big Data Within Healthcare Services: A Systematic Review.” Health Information Management Journal 54, no. 2: 190–201. 10.1177/18333583241270484. 39166442

[jnu70130-bib-0034] Kinchin, I. , E. Boland , I. Leroi , and J. Coast . 2025. “Through Their Eyes: Defining ‘Good Life’ in Dementia for Health Economics and Outcomes Research.” Social Science & Medicine 366: 117716. 10.1016/j.socscimed.2025.117716. 39837081

[jnu70130-bib-0035] Kleinheksel, A. J. , N. Rockich‐Winston , H. Tawfik , and T. R. Wyatt . 2020. “Demystifying Content Analysis.” American Journal of Pharmaceutical Education 84, no. 1: 7113. 10.5688/ajpe7113. 32292185 PMC7055418

[jnu70130-bib-0036] Lerum, S. V. , K. N. Solbraekke , and J. C. Frich . 2017. “Healthcare Professionals' Accounts of Challenges in Managing Motor Neurone Disease in Primary Healthcare: A Qualitative Study.” Health & Social Care in the Community 25, no. 4: 1355–1363. 10.1111/hsc.12432. 28226395

[jnu70130-bib-0037] Lorenz, K. A. , L. R. Shugarman , and J. Lynn . 2006. “Health Care Policy Issues in End‐Of‐Life Care.” Journal of Palliative Medicine 9, no. 3: 731–748. 10.1089/jpm.2006.9.731. 16752979

[jnu70130-bib-0038] Mahmood, S. , S. Law , and Y. Bombard . 2022. ““I Have to Start Learning How to Live With Becoming Sick”: A Scoping Review of the Lived Experiences of People With Huntington's Disease.” Clinical Genetics 101, no. 1: 3–19. 10.1111/cge.14024. 34216010

[jnu70130-bib-0039] Mitchell, G. , J. Agnelli , J. McGreevy , et al. 2016. “Palliative and End‐Of‐Life Care for People Living With Dementia in Care Homes: Part 2.” Nursing Standard 30, no. 44: 54–63. 10.7748/ns.2016.e10582. 27353937

[jnu70130-bib-0040] Naeem, M. , W. Ozuem , K. Howell , and S. Ranfagni . 2023. “A Step‐By‐Step Process of Thematic Analysis to Develop a Conceptual Model in Qualitative Research.” International Journal of Qualitative Methods 22: 16094069231205789. 10.1177/16094069231205789.

[jnu70130-bib-0041] National Institute for Health and Care Excellence . 2017. “Parkinson's Disease in Adults.” https://www.nice.org.uk/guidance/ng71/resources/parkinsons‐disease‐in‐adults‐pdf‐1837629189061.

[jnu70130-bib-0042] National Institute for Health and Care Excellence . 2019. “Motor Neurone Disease: Assessment and Management.” 32134605

[jnu70130-bib-0043] National Institute for Health and Care Excellence (NICE) . 2014. “Interim Methods Guide for Developing Service Guidance.” https://www.nice.org.uk/process/pmg8/chapter/appendix‐2‐checklists. 28230953

[jnu70130-bib-0044] National Institute for Health and Clinical Excellence/Social Care Institute for Excellence . 2018. Dementia: Assessment, Management and Support for People Living With Dementia and Their Carers. NICE Guideline 97. Methods, Evidence and Recommendationd. National Institute for Health and Care Excellence. 30011160

[jnu70130-bib-0045] North West Coast Strategic Clinical Network . 2018. “Palliative Care Guidelines in Dementia 2nd Edition.” https://www.england.nhs.uk/north/wp‐content/uploads/sites/5/2018/06/palliative‐care‐guidelines‐in‐dementia.pdf.

[jnu70130-bib-0046] Palliative Care Australia . 2023. “What is Palliative Care?—Palliative Care Australia.” https://palliativecare.org.au/resource/what‐is‐palliative‐care/.

[jnu70130-bib-0047] Palliative Care Australia & Neurological Alliance Australia . 2015. “Palliative Care and Neurological Conditions Position Statement.” https://palliativecare.org.au/wp‐content/uploads/dlm_uploads/2015/04/Neurological‐Position‐Statement‐PDF.pdf.

[jnu70130-bib-0048] Perez, E. , P. B. Perrin , S. K. Lageman , T. Villaseñor , and J. M. Dzierzewski . 2022. “Sleep, Caregiver Burden, and Life Satisfaction in Parkinson's Disease Caregivers: A Multinational Investigation.” Disability and Rehabilitation 44, no. 10: 1939–1945. 10.1080/09638288.2020.1814878. 32915084 PMC7947017

[jnu70130-bib-0049] Pickett, T. , E. Altmaier , and J. S. Paulsen . 2007. “Caregiver Burden in Huntington's Disease.” Rehabilitation Psychology 52, no. 3: 311–318. 10.1037/0090-5550.52.3.311.

[jnu70130-bib-0050] Rabins, P. V. , B. W. Rovner , T. Rummans , L. S. Schneider , and P. N. Tariot . 2017. “Practice Guideline for the Treatment of Patients With Alzheimer's Disease and Other Dementias.” Focus 15, no. 1: 110–128. 10.1176/appi.focus.15106. 31997970 PMC6519627

[jnu70130-bib-0051] Saunders, C. H. , A. Sierpe , C. Plessen , et al. 2023. “Practical Thematic Analysis: A Guide for Multidisciplinary Health Services Research Teams Engaging in Qualitative Analysis.” BMJ 381: e074256. 10.1136/bmj-2022-074256. 37290778

[jnu70130-bib-0052] Scottish Government . 2017. “Scotland's National Dementia Strategy 2017–2020.” https://www.gov.scot/binaries/content/documents/govscot/publications/strategy‐plan/2015/12/strategic‐framework‐action‐palliative‐end‐life‐care/documents/supporting‐evidence‐summary/supporting‐evidence‐summary/govscot%3Adocument/00491390.pdf.

[jnu70130-bib-0053] Scottish Government . 2023. “Dementia in Scotland: Everyone's Story.” https://www.gov.scot/binaries/content/documents/govscot/publications/strategy‐plan/2023/05/new‐dementia‐strategy‐scotland‐everyones‐story/documents/dementia‐scotland‐everyones‐story/dementia‐scotland‐everyones‐story/govscot%3Adocument/dementia‐scotland‐everyones‐story.pdf.

[jnu70130-bib-0054] Shoesmith, C. , A. Abrahao , T. Benstead , et al. 2020. “Canadian Best Practice Recommendations for the Management of Amyotrophic Lateral Sclerosis.” Canadian Medical Association Journal 192, no. 46: E1453–E1468. 10.1503/cmaj.191721. 33199452 PMC7683000

[jnu70130-bib-0055] Solari, A. , A. Giordano , J. Sastre‐Garriga , et al. 2020. “EAN Guideline on Palliative Care of People With Severe, Progressive Multiple Sclerosis.” Journal of Palliative Medicine 23, no. 11: 1426–1443. 10.1089/jpm.2020.0220. 32469284 PMC7583337

[jnu70130-bib-0056] Swiss Academy of Medical Sciences . 2018a. “Medical‐Ethical Guidelines: Care and Treatment of People With Dementia.” Swiss Medical Weekly 148: w14663. 10.4414/smw.2018.14663. 30428121

[jnu70130-bib-0057] Swiss Academy of Medical Sciences . 2018b. Care and Treatment of People With Dementia. Swiss Academy of Medical Sciences. 10.4414/smw.2018.14663 30428121

[jnu70130-bib-0058] Teoli, D. , C. Schoo , and V. B. Kalish . 2025. “Palliative Care.” In StatPearls. StatPearls Publishing. http://www.ncbi.nlm.nih.gov/books/NBK537113/. 30725798

[jnu70130-bib-0059] The Irish Palliative Care in Parkinson's Disease Group . 2016. Palliative Care in People With Parkinson's Disease: Guidelines for Professional Healthcare Workers on the Assessment and Management of Palliative Care Needs in Parkinson's Disease and Related Parkinsonian Syndromes. Univeristy College Cork. https://hospicefoundation.ie/wp‐content/uploads/2021/01/Palliative‐Care‐in‐people‐with‐Parkinsons‐disease.pdf.

[jnu70130-bib-0060] The Ministry of Health and Welfare . 2016. The 3rd National Dementia Plan: Living Well With Dementia in the Community. Ministry of Health and Welfare.

[jnu70130-bib-0061] The World Bank . 2025. “World Bank Country and Lending Groups. Country Classification.” https://datahelpdesk.worldbank.org/knowledgebase/articles/906519‐world‐bank‐country‐and‐lending‐groups.

[jnu70130-bib-0062] Tönges, L. , C. Buhmann , C. Eggers , S. Lorenzl , and T. Warnecke . 2024. “Guideline “Parkinson's Disease” of the German Society of Neurology (Deutsche Gesellschaft für Neurologie): Concepts of Care.” Journal of Neurology 271, no. 12: 7377–7386. 10.1007/s00415-024-12546-3. 38969876 PMC11588773

[jnu70130-bib-0063] Tyndall, J. 2010. AACODS Checklist. Flinders University. https://policycommons.net/artifacts/4855940/untitled/5692885/.

[jnu70130-bib-0064] Urushitani, M. , H. Warita , N. Atsuta , et al. 2024. “The Clinical Practice Guideline for the Management of Amyotrophic Lateral Sclerosis in Japan.” Rinshō Shinkeigaku 64, no. 4: 252–271. 10.5692/clinicalneurol.cn-001946. 38522911

[jnu70130-bib-0065] Vears, D. F. , and L. Gillam . 2022. “Inductive Content Analysis: A Guide for Beginning Qualitative Researchers.” Focus on Health Professional Education: A Multi‐Professional Journal 23, no. 1: 111–127. 10.11157/fohpe.v23i1.544.

[jnu70130-bib-0066] Vucic, S. , N. Shahrizaila , O. Kano , et al. 2025. “Pan‐Asian Consortium for Treatment and Research in ALS (PACTALS) Guidelines for Management of Amyotrophic Lateral Sclerosis.” Lancet Regional Health—Western Pacific 62: 101684. 10.1016/j.lanwpc.2025.101684. 41018945 PMC12465052

[jnu70130-bib-0067] Wang, C. , P. Song , and Y. Niu . 2022. “The Management of Dementia Worldwide: A Review on Policy Practices, Clinical Guidelines, End‐Of‐Life Care, and Challenge Along With Aging Population.” Bioscience Trends 16, no. 2: 119–129. 10.5582/bst.2022.01042. 35466154

[jnu70130-bib-0068] Weafer, J. A. 2014. The Palliative Care Needs of People With Advancing Neurological Disease in Ireland. Irish Hospice Foundation.

[jnu70130-bib-0069] Williams, M. , and T. Moser . 2019. “The Art of Coding and Thematic Exploration in Qualitative Research.” International Management Review 15, no. 1: 45–55.

[jnu70130-bib-0070] Wilson, E. , J. Seymour , and A. Aubeeluck . 2011. “Perspectives of Staff Providing Care at the End of Life for People With Progressive Long‐Term Neurological Conditions.” Palliative & Supportive Care 9, no. 4: 377–385. 10.1017/S1478951511000393. 22104413

[jnu70130-bib-0071] World Health Organization . 2004. Better Palliative Care for Older People. WHO Regional Office for Europe. https://iris.who.int/server/api/core/bitstreams/131c8820‐fd88‐4034‐8d87‐916ea2bc69d1/content.

[jnu70130-bib-0072] World Health Organization (WHO) . 2020. Palliative Care. World Health Organization. https://www.who.int/health‐topics/palliative‐care.

[jnu70130-bib-0073] World Health Organization (WHO) . n.d. “Palliative Care: The Essential Facts.” https://cdn.who.int/media/docs/default‐source/integrated‐health‐services‐(ihs)/palliative‐care/palliative‐care‐essential‐facts.pdf?sfvrsn=c5fed6dc_1.

[jnu70130-bib-0074] Xiong, B. , D. X. Bailey , P. Prudon , et al. 2024. “Identification and Information Management of Cognitive Impairment of Patients in Acute Care Hospitals: An Integrative Review.” International Journal of Nursing Sciences 11, no. 1: 120–132. 10.1016/j.ijnss.2023.11.001. 38352291 PMC10859579

