# Supplementary material for: A Systematic Review of Worldwide Guidelines, Protocols, and Health Care Standards for Provision of Palliative Care Services in Progressive Neurological Disorders
Source: J Nurs Scholarsh. 2026 Sep 8;58(5):e70130. doi: 10.1111/jnu.70130 (PMC13551532; doi:10.1111/jnu.70130)
Supplement: Supplementary file 1 [file JNU-58-0-s001.docx]

## Supplementary Table 1: Search terms

| 1 | guideline*.mp. [mp=title, book title, abstract, original title, name of substance word, subject heading word, floating sub-heading word, keyword heading word, organism supplementary concept word, protocol supplementary concept word, rare disease supplementary concept word, unique identifier, synonyms, population supplementary concept word, anatomy supplementary concept word] |
| --- | --- |
| 2 | (policy or policies).mp. [mp=title, book title, abstract, original title, name of substance word, subject heading word, floating sub-heading word, keyword heading word, organism supplementary concept word, protocol supplementary concept word, rare disease supplementary concept word, unique identifier, synonyms, population supplementary concept word, anatomy supplementary concept word] |
| 3 | ((clinical or care or nursing) adj protocol*).mp. [mp=title, book title, abstract, original title, name of substance word, subject heading word, floating sub-heading word, keyword heading word, organism supplementary concept word, protocol supplementary concept word, rare disease supplementary concept word, unique identifier, synonyms, population supplementary concept word, anatomy supplementary concept word] |
| 4 | best practice.mp. [mp=title, book title, abstract, original title, name of substance word, subject heading word, floating sub-heading word, keyword heading word, organism supplementary concept word, protocol supplementary concept word, rare disease supplementary concept word, unique identifier, synonyms, population supplementary concept word, anatomy supplementary concept word] |
| 5 | (palliative or palliation or end of life care or care of the dying or terminal care or hospice care).mp. [mp=title, book title, abstract, original title, name of substance word, subject heading word, floating sub-heading word, keyword heading word, organism supplementary concept word, protocol supplementary concept word, rare disease supplementary concept word, unique identifier, synonyms, population supplementary concept word, anatomy supplementary concept word] |
| 6 | neurodegen*.mp. [mp=title, book title, abstract, original title, name of substance word, subject heading word, floating sub-heading word, keyword heading word, organism supplementary concept word, protocol supplementary concept word, rare disease supplementary concept word, unique identifier, synonyms, population supplementary concept word, anatomy supplementary concept word] |
| 7 | (progressive neurological adj (condition* or disease* or disorder*)).mp. [mp=title, book title, abstract, original title, name of substance word, subject heading word, floating sub-heading word, keyword heading word, organism supplementary concept word, protocol supplementary concept word, rare disease supplementary concept word, unique identifier, synonyms, population supplementary concept word, anatomy supplementary concept word] |
| 8 | Alzheimer*.mp. [mp=title, book title, abstract, original title, name of substance word, subject heading word, floating sub-heading word, keyword heading word, organism supplementary concept word, protocol supplementary concept word, rare disease supplementary concept word, unique identifier, synonyms, population supplementary concept word, anatomy supplementary concept word] |
| 9 | Dementia.mp. [mp=title, book title, abstract, original title, name of substance word, subject heading word, floating sub-heading word, keyword heading word, organism supplementary concept word, protocol supplementary concept word, rare disease supplementary concept word, unique identifier, synonyms, population supplementary concept word, anatomy supplementary concept word] |
| 10 | motor neurone disease.mp. [mp=title, book title, abstract, original title, name of substance word, subject heading word, floating sub-heading word, keyword heading word, organism supplementary concept word, protocol supplementary concept word, rare disease supplementary concept word, unique identifier, synonyms, population supplementary concept word, anatomy supplementary concept word] |
| 11 | amyotrophic lateral sclerosis.mp. [mp=title, book title, abstract, original title, name of substance word, subject heading word, floating sub-heading word, keyword heading word, organism supplementary concept word, protocol supplementary concept word, rare disease supplementary concept word, unique identifier, synonyms, population supplementary concept word, anatomy supplementary concept word] |
| 12 | Huntington* disease.mp. [mp=title, book title, abstract, original title, name of substance word, subject heading word, floating sub-heading word, keyword heading word, organism supplementary concept word, protocol supplementary concept word, rare disease supplementary concept word, unique identifier, synonyms, population supplementary concept word, anatomy supplementary concept word |
| 13 | Parkinson* disease.mp. [mp=title, book title, abstract, original title, name of substance word, subject heading word, floating sub-heading word, keyword heading word, organism supplementary concept word, protocol supplementary concept word, rare disease supplementary concept word, unique identifier, synonyms, population supplementary concept word, anatomy supplementary concept word] |
| 14 | 1 or 2 or 3 or 4 |
| 15 | 6 or 7 or 8 or 9 or 10 or 11 or 12 or 13 |
| 16 | 5 and 14 and 15 |

## Supplementary table 2: Characteristics of the guidelines

| **Country / Region of origin** | **N (%)** |
| --- | --- |
| Australia | 5 (14) |
| UK | 6 (17) |
| Ireland | 3 (9) |
| Switzerland | 3 (9) |
| Canada | 3 (9) |
| Europe | 3 (9) |
| Japan | 1 (3) |
| Scotland | 2 (6) |
| USA | 1 (3) |
| Germany | 1(3) |
| Italy | 1 (3) |
| Iran | 1 (3) |
| Korea | 1 (3) |
| Multinational | 2 (6) |
| **Progressive neurological condition(s)** | **n (%)** |
| Dementia | 18 (49) |
| Multiple Sclerosis | 1 (3) |
| Amyotrophic lateral sclerosis | 5 (14) |
| General PND or PND and other non-PND | 4 (11) |
| Parkinson’s disease | 4 (11) |
| Panthothenate kinase-associated neurodegeneration (PKAN) | 1 (3) |

## Supplementary Table 3: AACODS Checklist

| **AACODS** |  | **Yes** | **No** | **?** |
| --- | --- | --- | --- | --- |
| **Authority** | Identifying who is responsible for the intellectual content.  **Individual author:**   - Associated with a reputable organisation? - Professional qualifications or considerable experience? - Produced/published other work (grey/black) in the field? - Recognised expert, identified in other sources? - Cited by others? (use Google Scholar as a quick check) - Higher degree student under “expert” supervision?   **Organisation or group:**   - Is the group reputable? (e.g. W.H.O) - Is the organisation an authority in the field?   **In all cases:**   - Does the item have a detailed reference list or biography? |  |  |  |
| **Accuracy** | - Does the item have a clearly stated aim or brief? - If so, is this met? - Does it have a stated methodology? - If so, is it adhered to? - Has it been peer-reviewed? - Has it been edited by a reputable authority? - Supported by authoritative, documented references or credible sources? - Is it representative of work in the field? - If No, is it a valid counterbalance? - Is any data collection explicit and appropriate for the research? - If item is secondary material (e.g. a policy brief or a technical report) refer to the original - Is it an accurate, unbiased interpretation or analysis? |  |  |  |
| **Coverage** | All items have parameters which define their content coverage. These limits might mean that a work refers to a particular population group, or that it excluded certain types of publication. A report could be designed to answer a particular question, or be based on statistics from a particular survey.   - Are any limits clearly stated? |  |  |  |
| **Objectivity** | It is important to identify bias, particularly if it is unstated or unacknowledged.   - Opinion, expert or otherwise, is still opinion: is the author’s standpoint clear? - Does the work seem to be balanced in presentation? |  |  |  |
| **Date** | For the item to inform your research, it needs to have a date that confirms relevance.   - Does the item have a clearly stated date related to content? No easily discernible date is a strong concern. - If no date is given, but can be closely ascertained, is there a valid reason for its absence? - Check the bibliography: have key contemporary material been included? |  |  |  |
| **Significance** | There is a value judgement of the item, in the context of the relevant research area   - Is the item meaningful? (this incorporates feasibility, utility, and relevance) - Does it add context? - Does it enrich or add something unique to the research? - Does it strengthen or refute a current position? - Would the research area be lesser without it? - Is it integral, representative, typical? - Does it have impact? (in the sense of influencing the work or behaviour of others) |  |  |  |
